# γ irradiation induced effects on bismuth active centres and related photoluminescence properties of Bi/Er co-doped optical fibres

**DOI:** 10.1038/srep29827

**Published:** 2016-07-21

**Authors:** D. Sporea, L. Mihai, D. Neguţ, Yanhua Luo, Binbin Yan, Mingjie Ding, Shuen Wei, Gang-Ding Peng

**Affiliations:** 1 National Institute for Laser, Plasma and Radiation Physics, Laser Metrology Laboratory, Măgurele, RO-077125, Romania; 2 “Horia Hulubei” National Institute of Physics and Nuclear Engineering, IRASM Radiation Processing Department, RO-077125, Măgurele, Romania; 3 Photonics & Optical Communications, School of Electrical Engineering & Telecommunications, University of New South Wales, Sydney, NSW 2052, Australia; 4 State Key Laboratory of Information Photonics and Optical Communications, Beijing University of Posts and Telecommunications, Beijing 100876, China

## Abstract

We investigate the effects of γ irradiation on bismuth active centres (BACs) and related photoluminescence properties of bismuth/erbium co-doped silica fibre (BEDF), [Si] ~28, [Ge] ~1.60, [Al] ~0.10, [Er] ~ <0.10 and [Bi] ~0.10 atom%, fabricated by *in-situ* solution doping and Modified Chemical Vapor Deposition (MCVD). The samples were irradiated at 1 kGy, 5 kGy, 15 kGy, 30 kGy and 50 kGy doses, and dose rate of 5.5 kGy/h, at room temperature. The optical properties of BEDF samples are tested before and after γ irradiation. We found that high dose γ irradiation could significantly influence the formation and composition of BACs and their photoluminescence performance, as important changes in absorption and emission properties associated with the 830 nm pump produces the direct evidence of γ irradiation effects on BAC-Si. We notice that the saturable to unsaturable absorption ratio at pump wavelength could be increased with high dose γ irradiation, indicating that emission and pump efficiency could be increased by γ irradiation. Our experimental results also reveal good radiation survivability of the BEDF under low and moderate γ irradiation. Our investigation suggests the existence of irradiation related processing available for tailoring the photoluminescence properties and performance of bismuth doped/co-doped fibres.

For more than 15 years, the use of Erbium Doped (or co-doped) Fibre Amplifier (EDFA) in space-borne missions was considered for inter-satellite communications and fibre optic gyroscopes (FOGs) applications^1^,^2^,^3^, as components deployed on the board of space missions have to face exposure to electrons and protons trapped in the Van Allen belts, heavy ions trapped in the magnetosphere, protons and heavy ions associated to cosmic ray or solar flares^4^. Several tests were run in order to assess the degradation of doped optical fibres under ionizing radiation. One such investigation focused on gamma irradiation (total dose 300 Gy, maximum dose rate 45 Gy[H_2_O]/h) of optical fibres produced by Surface Plasma Chemical Vapor Deposition (SPCVD) process, having the cladding of pure silica and the core doped with nitrogen and erbium^1^. The radiation-induced absorption (RIA) proved to be higher at the pump wavelength (960 nm) than that at the signal wavelength (1560 nm)^1^.

In another approach, one of the tested optical fibre was doped in the core with Er and F, while the other had in the core only Al^2^. The radiation sensitivity of the Er-doped optical fibre increases with the Er concentration, for all the dose rates used^1.2^. The inclusion of Al induced a RIA in the infrared (IR) of the same order of magnitude as Er^2^.

One extensive study indicated that the RIA on rare earth-doped optical fibres (Er or Yb) is less dependent on the main dopant and is more influenced by the co-dopants concentration (Al, Ge, P) and the manufacturing process^3^. The radiation induced effects depend on the total dose used and less on the type of radiation (proton, neutron, gamma rays) to which the samples were exposed. By heating the irradiated optical fibres up to 450 K a recovery of RIA was obtained, to 70% of the initial transmission.

In another experiment six commercially available EDFs 3 m long, spliced with SMF-28 leads and spooled at 7.5 to 10 cm diameter were exposed to gamma radiation (dose rate 0.014 Gy/s, total dose 500 Gy). Two of the optical fibres were irradiated with protons with the energies of 5.6 and 28 MeV (at the fluences in the fibre core of 4.3 × 10^10^/cm^2^ and 1.9 × 10^11^/cm^2^), up to the dose of 500 Gy. The optical fibres spectral transmission was recorded over the 800 nm–1700 nm spectral range^5^. The optical gain and the noise function vs. the pump power characteristics, monitored during gamma irradiation, showing a saturation effect dependent on the optical fibre type and the total dose. The spectral attenuation is more significant below 1100 nm. The degradation of the amplifiers parameters seems to be attributed to the increase of the absorption at the pump wavelength.

Irradiation of an EDF source (EDFS) up to the total dose of 33.5 krad (Si), with a dose rate of 6 rad/min produced both the decrease of the output power and the change of its spectral distribution^6^.

Two Er and Al co-doped commercially available optical fibers were subjected to gamma irradiation (at a dose rate of 530 Gy/h, to a total dose of 3.18 kGy) with one of the optical fibres having a high Al concentration. After the irradiation the two optical fibres were pumped with a tunable fs laser at around 810 nm and the 1550 nm fluorescence was monitored in real time^7^. The conclusions of this investigation are: (i) the higher Al concentration makes the optical fibre more vulnerable to gamma irradiation; (ii) the fluorescence signal at 1550 nm is almost recovered after pumping with the fs laser radiation, while the fluorescence emission of the gamma exposed optical fibre with a higher Al content did not recovered completely, it saturated in about 1000 s.

For the case of Er/Yb optical fibres having different co-dopant concentrations a linear dependence of the losses at 1310 nm with the total dose was observed. Also, it was noticed that the dose rate plays an important role in the increase of the attenuation, as higher dose rates lead to higher losses^8^.

Yb-doped and Yb/Er-doped optical fibres (YbP, YbAl, YbPAl, YbErP, YbErPAl) were irradiated by 10 keV X-rays, 1 MeV gamma rays and 105 MeV protons, and the radiation induced effects were studied by optical absorption (from 850 nm to 1700 nm), confocal microscopy of luminescence, and by the excitation of the fibre core with radiation from a supercontinuum light source^9^. RIA at 1550 nm is comparable for gamma-rays and proton exposure. Al and P dopants increase the radiation sensitivity.

During an investigation run under gamma irradiation (dose rates from 0.14 Gy/s(Si) to 1.2 Gy/s(Si), up to the total dose of 1.5 kGy) on five single- and double-clad, highly Yb-doped aluminosilicate optical fibres from Liekki a decrease of the optical transmission over the spectral range 1100 nm to 1650 nm was observed^10^.

Yb^3+^ doped silica glass preforms having different concentrations of P and Al were exposed to gamma-rays and investigated using Raman scattering, optical absorption and Electron Spin Resonance (ESR) to assess the changes^11^. The experiment indicated that a high P concentration provides a resistance of the optical material to photodarkening, while in the case the Al concentration is higher, a large absorption band appears.

The influence of H_2_-loading of EDFs on the efficiency by pumping at 980 nm and 1480 nm was investigated by gamma irradiating, with doses between 0.1 and 10 kGy, of one H_2_-loaded and one H_2_-free carbon-coated EDF^12^. By pumping at 980 nm a photobleaching effect was observed which increases the efficiency of the process especially in the H_2_-loaded optical fibre. Apart from H_2_-loading some other methods were used to obtain radiation hardened optical fibres for optical amplifiers, such as: Ce doping of the fibre core^13^,^14^ and Hole-Assisted Carbon-Coated pre-treated with D_2_^15^.

The enhancement of photoluminescence in gamma irradiated Bi/Al co-doped silica optical fibers was recently reported^16^.

The point defect plays an important role in the formation of the bismuth related active centres (BACs). A recent report has confirmed that Bi-related NIR emitting centers consist of Bi ions and oxygen deficiency centers (ODC) but not Bi ions themselves^17^.

The role of the co-dopants in the radiation response of the fibres remains largely unclear so far^11^,^18^. Previously, the evidence of AlOHC responsible for the radiation-induced darkening in Yb doped fibre has been reported after characterizing a set of γ-irradiated Yb^3+^ doped silica glass preforms with different contents of phosphorous and aluminium^11^. Interestingly they found that, when there is more P than Al, phosphorous formed a cage (or solvation shell) surrounding Yb^3+^. They demonstrate that when P is introduced in excess compared to Al, nearly no radiodarkening is induced by γ-rays. On the other hand, when more Al than P is present, a large absorption band is induced by radiation. Thermal annealing experiments reveal the correlation between the decrease of the optical absorption band and the decrease of the Al-Oxygen Hole Center (AlOHC) ESR signal, demonstrating the main role of AlOHC defects in the fibre darkening. More recently, as reported in ref. 18, it is considered that the presence of Al ions is one reason for the Er^3+^ into Er^2+^ ion reduction after irradiation. Their explanation is that the vicinity of Al to the rare earth elements in many glasses is linked to the divalent rare earth species (Er^3+^, Yb^3+^) and AlOHC.

A new type of doped optical fibres, bismuth/erbium co-doped fibre (BEDF), aimed for optical fibre amplifiers was developed at UNSW. The particular feature of this optical fibre as compared to previously reported doped optical fibres is the ultra broadband emission (1100 nm to 1800 nm operating range)^19^.

Being a new material with a great potential for optical communications, we were interested to investigate how it performs under ionizing radiation, for its possible use in space-borne applications. The major focus of our research was related to the way radiation exposure affects the pumping conditions, the emission characteristics, as well as the overall light propagation performances in these fibres. By this study we also try to evaluate their reliability limits. In this paper we report for the first time the results of an extended study concerning the gamma-ray effects on BEDF optical fibre.

## Results

The tested samples are described under section “Methods: Materials”. The irradiation conditions are presented in section “Methods: Irradiation”. The description of the setups used for laboratory measurements is provided in section “Methods: Measurements”.

*For definitions of employed terms and notations see section “Methods: Measurements”. Data not included into the main body of the paper can be found under “*Supplementary materials*”. Figures and Tables to be found in this part of the work are cited in the main text accompanied by “S”.*

The small signal absorption (α @ 1300 nm), as well as the pump absorption coefficients: maximum pump absorption (α_max_), saturable pump absorption (α_s_), unsaturable pump absorption (α_us_) values measured before and after the irradiation, for the irradiation doses used, are given in Supplementary Table S1. The measuring conditions (i.e. wavelength to which the measuring setup was operated) is also specified.

**Figure 1:**
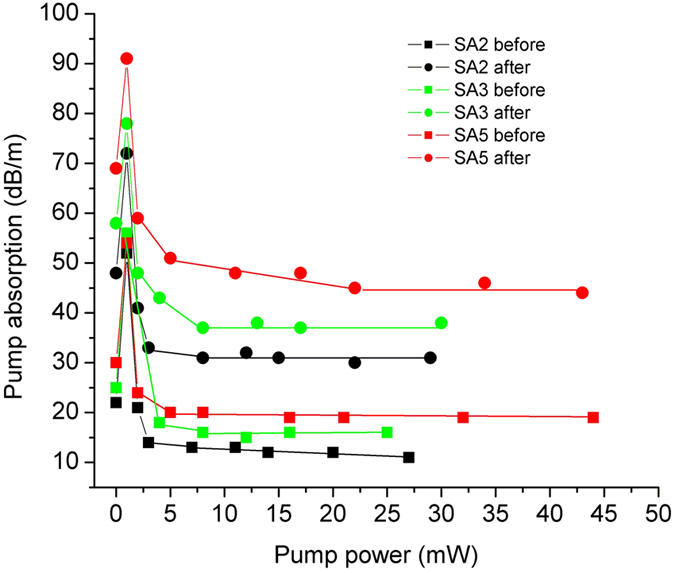
The effect of the total irradiation dose on pump absorption as function of the pump power at 830 nm: SA2, SA3 and SA5 before and after 5 kGy, 15 kGy and 50 kGy dose radiation, respectively.

**Figure 2:**
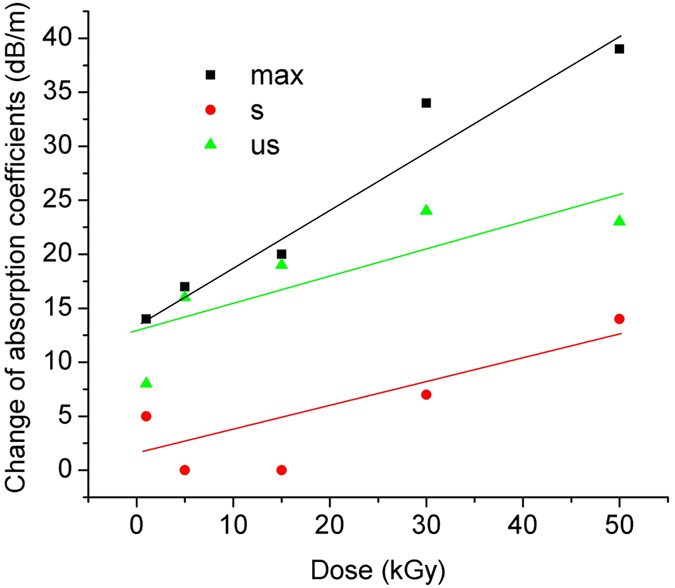
The variation of the pump absorption coefficients: *max* − Δα_max,_ *s* − Δα_s_, *us* − Δα_us_ with the total irradiation dose for the tested samples.

**Figure 3:**
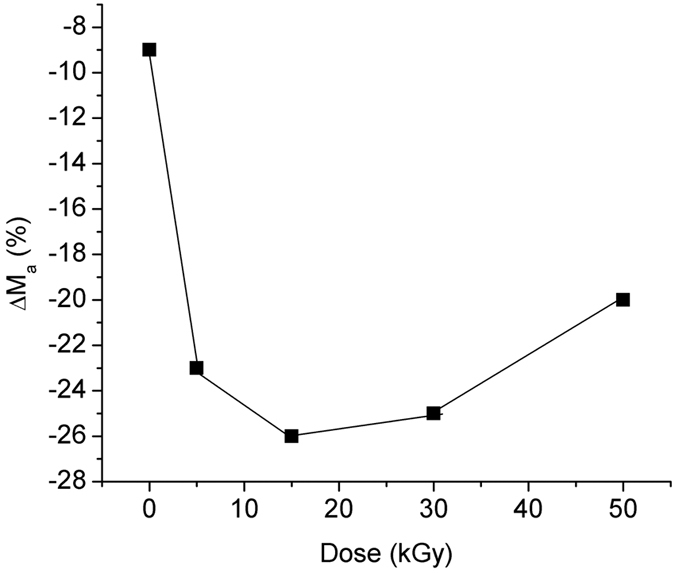
The figure of merit change ΔM_a_ at 830 nm pump vs. the irradiation dose.

We investigated for the irradiated samples the dependence of the pump total loss (T_t_), equation (3), on the pump power at λ = 830 nm, as illustrated in Fig. 1. The variation of the pump absorption coefficients α_max,_ α_s_, α_us_, equations (4)–(6), 5, 6, with the total irradiation dose is given in Fig. 2. The figure of merit change - ΔM_a_, equations (7) and (8), at 830 nm pump, with the dose is illustrated in Fig. 3.

**Figure 4:**
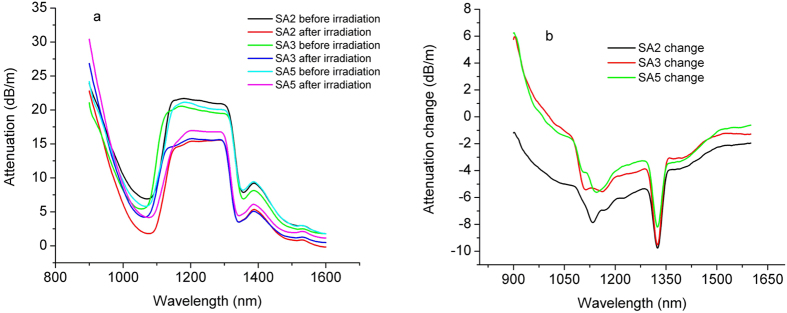
The attenuation spectra (**a**) and the attenuation change induced by gamma irradiation (**b**) of samples SA2, SA3 and SA5 before and after gamma irradiation at 5 kGy, 15 kGy and 50 kGy dose, respectively.

The total spectral attenuation was measured for the pristine optical fibre and for each irradiation dose. The results for the total doses of 5 kGy, 15 kGy and 50 kGy are presented in Fig. 4a. In Fig. 4b we illustrate the difference between the optical attenuation before and after the irradiation as induced in samples SA2, SA3 and SA5. The measurements were run over the 800 nm to 1600 nm spectral range. For irradiation details, see section “Methods: Irradiation”.

**Figure 5:**
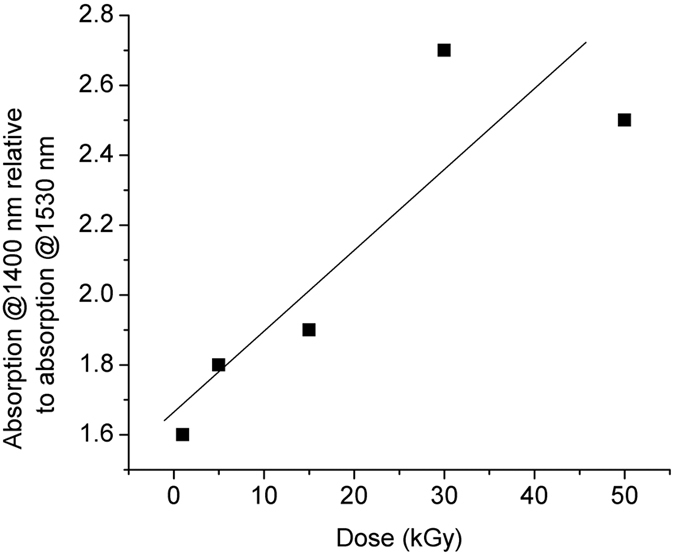
The change of the absorption at 1400 nm referred to the absorption change at 1530 nm vs. the irradiation dose.

In Fig. 5 we provide information on the relative change of the attenuation at 1400 nm referred to the value for 1530 nm, for the five values of the irradiation dose.

Other parameters of interest we studied in relation to gamma irradiation are: the backward – e_1_(λ), and forward – e_2_(λ) propagated spectral emission. Supplementary Table S2 represents a synopsis of the tests carried out in relation to the BEDF emission pre and post irradiation, results detailed in the following section. e(λ) designates the spectral variation, while e_2_(1100), for example, represents the forward emission measured at λ = 1100 nm. P_sat_ denotes the saturation power.

**Figure 6:**
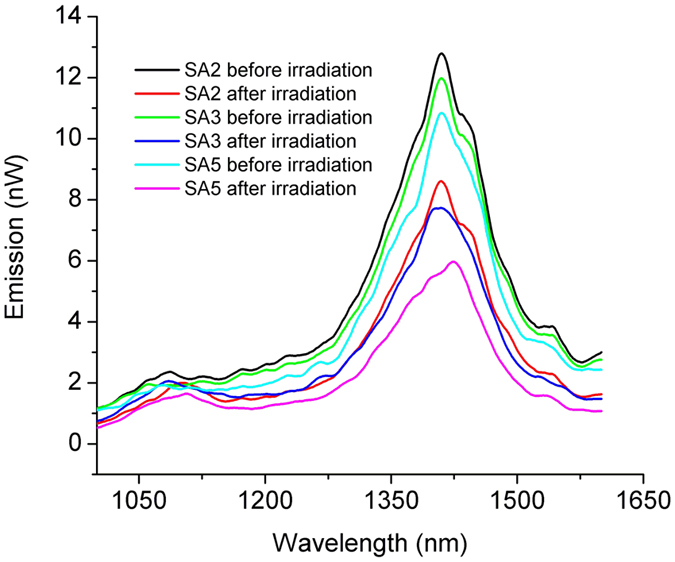
The forward e_2_(λ) propagated spectral emission for sample SA2, SA3 and SA5 before and after 5 kGy, 15 kGy and 50 kGy gamma irradiation.

The forward emission before and after 5 kGy, 15 kGy and 50 kGy gamma radiation are depicted in Fig. 6. The dependence of the backward and forward emissions on the pump power at 830 nm, before and after 1 kGy gamma exposure, is illustrated in Supplementary Fig. S1. The values represented in Fig. 6 are corrected against the background signal, and the value of the pump power at which the test was run is specified in Supplementary Table S3.

**Figure 7:**
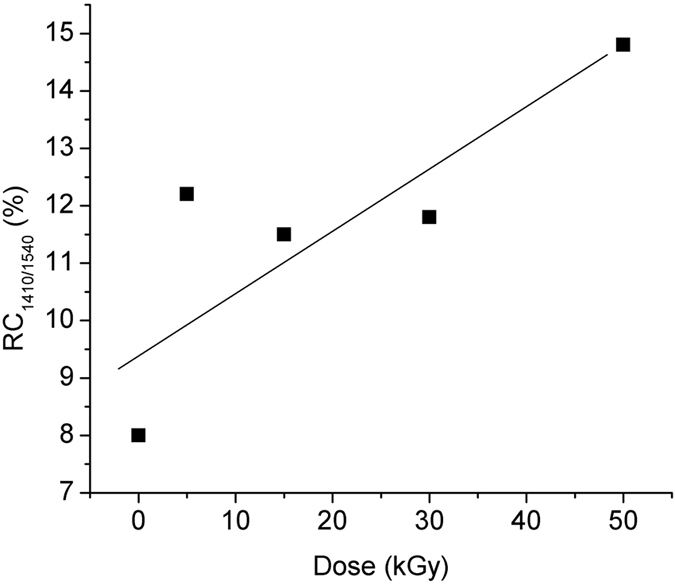
The relative change of the ratio forward emission at 1410 nm and forward emission at 1540 nm induced by gamma radiation vs. the irradiation dose.

The relative change of the ratio of the forward emission at 1410 nm referred to the forward emission at 1540 nm – RC_1410/1540_, equations (9) and (12), as function of the irradiation dose is illustrated in Fig. 7.

**Figure 8:**
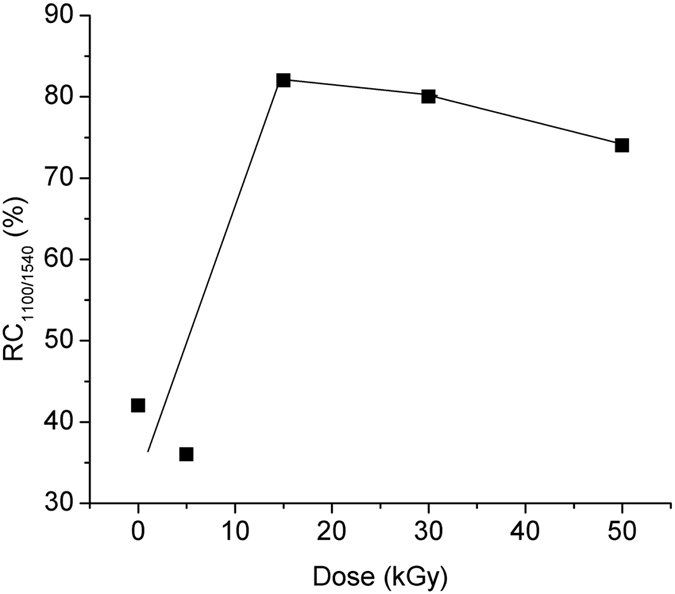
The relative change of the ratio of the forward emission at 1100 nm to the forward emission at 1540 nm induced by gamma radiation vs. the irradiation dose.

The relative change of the ratio of the forward emission at 1100 nm referred to the forward emission at 1540 nm – RC_1100/1540_, equations (10) and (13), as function of the irradiation dose, is illustrated in Fig. 8.

**Figure 9:**
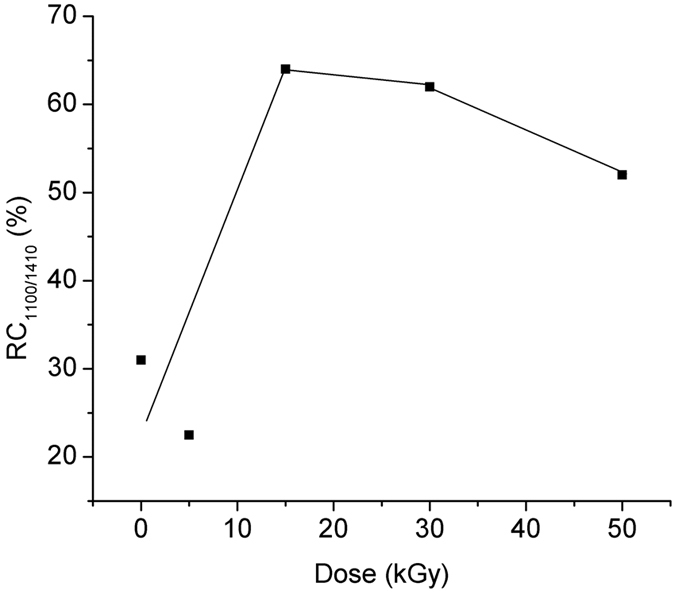
The relative change of the ratio of the forward emission at 1100 nm to the forward emission at 1410 nm induced by gamma radiation vs. dose.

The relative change of the ratio of the forward emission at 1100 nm referred to the forward emission at 1410 nm – RC_1100/1410_, equations (11) and (14), as function of the irradiation dose is illustrated in Fig. 9.

**Figure 10:**
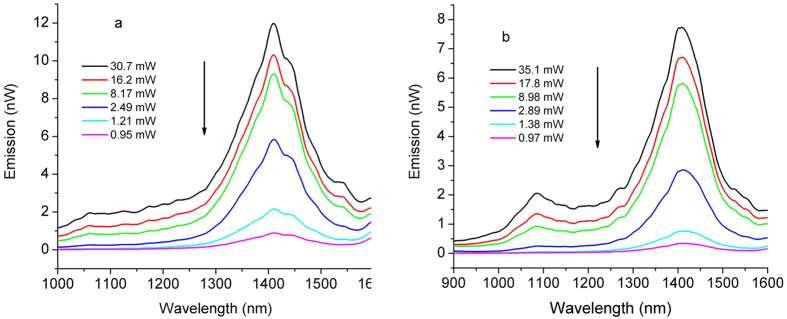
Emission spectra of SA3 before (**a**) and after (**b**) 15 kGy gamma radiation under different pump power.

Another evaluation was carried out in relation to the effect of gamma irradiation on the spectral behaviour of the forward emission for different pump power. In Fig. 10 are presented the results for sample SA3 before (a) and after (b) 15 kGy gamma exposure.

**Figure 11:**
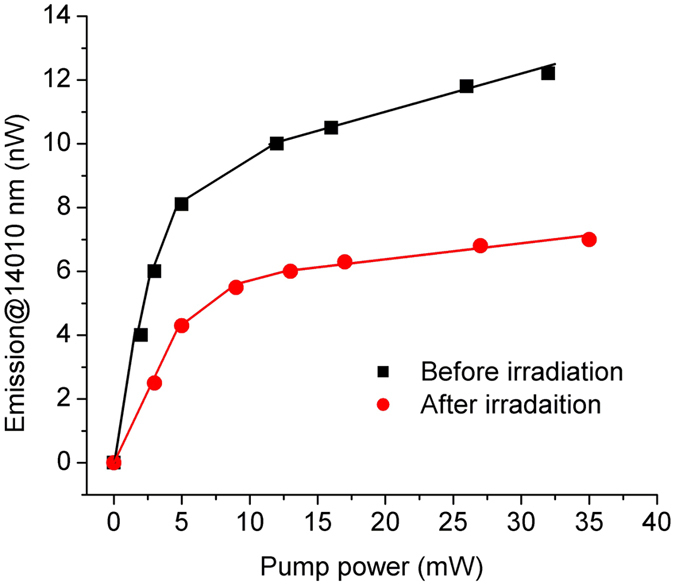
Emission at 1410 nm for sample SA3 before and after 15 kGy gamma radiation vs. the pump power.

The dependence of the forward emission on the pump power, at λ = 1410 nm, before and after gamma irradiation is illustrated in Fig. 11, for the total dose of 15 kGy.

**Figure 12:**
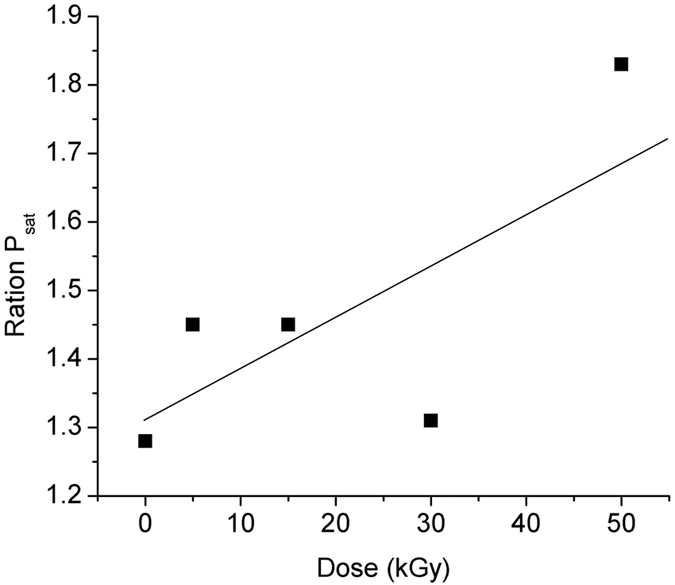
Ratio of saturation power before and after radiation vs. the irradiation dose.

In Fig. 12 we represented the change of the saturation power (P_sat_) as function of the total dose. The pump power required to reach half inversion (i.e. bleach the transition) is defined as the saturation power^20^.

## Discussion

Previous studies indicated for the investigated doped optical fibres that:

1. the radiation sensitivity increases with the Er and some co-dopants (i.e. Al, Ge, P) concentration;
2. exposure to ionizing radiation induces spectral transmission losses and a decrease of the output power;
3. the radiation effects are more dependent on the total dose used than the type of radiation involved;
4. higher dose rates produce higher attenuation in the fibre;
5. the presence of Bi in the composition of the optical fibre core has enhanced the photoluminescence upon gamma irradiation.

Within this context, we run our research as follows:

1. gamma irradiation without any heating during or post irradiation;
2. passive, off-line tests, to be able to evaluate through a complex investigation the post irradiation modification of the pump and emission conditions;
3. use of a quite high radiation dose, in order to be able to assess the radiation induced degradation for high total doses;
4. comparative measurements at wavelength associated to the BAC-Al and the BAC-Si (Bi active center – BAC), for the ingredients present along with Bi in the fibre core, to check the effect of gamma-rays on the emission corresponding to these centers.

Referring to the pump absorption dependence on the pump power at 830 nm (Fig. 1) can be noticed that the level of the pump power saturation is quite the same after 5 kGy and 15 kGy irradiation, but is shifted towards higher pump powers as the dose increases to 50 kGy.

The pump absorption coefficients show a linear increase with the total dose (Fig. 2), having radiation sensitivities α_RIA_ of: α_RIA_(α_max_) = 0.58 dB/(m∙kGy); α_RIA_(α_s_) = 0.22 dB/(m∙kGy); α_RIA_(α_us_) = 0.25 dB/(m∙kGy), sensitivities defined similar to RIA sensitivity^1^. All curve fittings were done with a confidence level of 95%. As indicated by the investigation results, the saturable to unsaturable absorption ratio at pump wavelength could be increased with high dose γ irradiation, indicating that emission and pump efficiency could be increased by γ irradiation if the BEDF parameters and pump conditions are optimized. The increase of the saturable pump absorption (α_s_), and the unsaturable pump absorption (α_us_) with the dose contribute to the change of the figure of merit (ΔM_a_) with the irradiation dose. Up to 15 kGy the ΔM_a_ can be described by the exponential decay (Fig. 3):


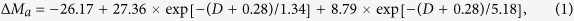


while for doses (D) higher than 15 kGy, the ΔM_a_ increase can be expressed by the exponential grow curve:


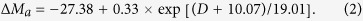


The analysis of the two equations leads to the conclusion that appropriate exposure to gamma radiation of the BEDF we propose can prevent, within some limits, the degradation of M_a_ upon irradiation, at λ = 830 nm.

The measurements carried out on the spectral optical attenuation (Fig. 4a,b) highlights that the BEDF exposure to gamma-rays increases the spectral transmission up to the dose of 15 kGy when a saturation effect occurs. This phenomenon uncommon to irradiated optical fibres can be attributed to the irradiation induced changes in BAC. An exception appears at λ = 1320 nm, where only high doses (i.e. 50 kGy) induce a decrease of the attenuation.

Figures 4a and 5 indicate that the RIA has lower values at 1400 nm than that at 1530 nm after the irradiation, fact which can be exploited by operating the fibre at the lower wavelength. This advantage appears to be a benefit even for the increase of the total dose. So, the BEDF proves to be more reliable at 1400 nm, as it can support high radiation exposure.

The photoluminescence signal at λ = 1400 nm degrades gradually with the dose increase (Fig. 6), while that corresponding to λ = 1100 nm remains almost unchanged after the irradiation. Nevertheless, the maximum emission after irradiation shifts towards high wavelengths (i.e. from λ = 1408 nm and 5 kGy dose to λ = 1423 nm and 50 kGy dose).

Figures 7, 8, 9 indicate that over some dose intervals specific wavelengths (i.e. λ = 1100 nm, λ = 1400 nm) are favoured by the irradiation, making them more suitable for operation under irradiation conditions. These effects can be explained by the activation of the associated centers (BAC-Al and BAC-Si) following the exposure to gamma radiation. As it concerns the emission at λ = 1100 nm, the ionizing radiation can improve the output signal with appropriate pump power applied (Fig. 10b).

As the dose increases (Fig. 12) the pump power required to reach half inversion increases, reflecting the effective quantity of BAC under these circumstances.

To conclude, our investigations on γ irradiated BEDFs indicated:

1. a good reliability of these fibres under low and medium irradiation doses, at quite high dose rates, recommending them for possible use in radiation environments;
2. an increase of the optical transmission over the spectral range 900 nm–1650 nm under γ irradiation up to the dose of 15 kGy;
3. that γ irradiation could increase the saturable to unsaturable absorption ratio at pump wavelength leading to an improve of the emission and pump efficiency;
4. that appropriate selection of the operating wavelength can be considered as an option for the operation of a radiation hardened device;
5. the existence of some design provisions (related to the fibre composition and γ dose) which can be exploited to improve the Bi doped/co-doped fibres performances and their photoluminescence characteristics.

The investigation outcomes provide evidence that by γ irradiation is possible to significantly influence the formation of relevant BACs or to change the BAC compositions.

## Methods

### Materials

The tested doped optical fibre was produced at UNSW by *in-situ* solution doping and MCVD techniques. The dopant concentrations in BEDF are measured to be: [Si] ~28, [Ge] ~1.60, [Al] ~0.10, [Er] ~ < 0.10 and [Bi] ~0.10 atom%, respectively. The incorporation of a certain amount of Al and P in silica will significantly increase the flexibility of glass network and prevent dopant clustering^21^,^22^. P and Al co-doping has been studied to optimize the composition and to prevent clustering in Yb^3+^-doped aluminosilicate, borosilicate and phosphate glasses^22^. The fibre has a NA ~0.235, a core diameter ~3.0 μm and a λ_c_ ~1.04 μm.

For the tests carried out pre and post gamma irradiation we used samples of BEDF spliced at one end to a piece of 1.2 m commercial single mode (SM) communication optical fibre, optimized for the operation at 1300 nm and designated as SMF @ 1300, and at the other end to a piece of 2 m of commercial SM communication optical fibre, optimized for operation at 1550 nm and denoted by SMF @ 1550. The two communication type optical fibers are not connectorized, and one such sample, as illustrated in Supplementary Fig. S2, is indicated in the paper as sample SA. Five SA samples were prepared to be subjected to five total gamma doses (Supplementary Table S4).

### Irradiation

The irradiation of the samples was done at the ^60^Co GC-5000 (BRIT, India) irradiator of the “Horia Hulubei” National Institute of Physics and Nuclear Engineering, having an irradiation chamber volume of 5000 cm^3^. For irradiation, all samples were concentrically spooled to a planar geometry with a diameter of about 8 cm, separately mounted between two cardboard sheets (Supplementary Fig. S3). The samples were placed in the middle of the cylindrical irradiation chamber, at the distance of 10 cm from the base. The maximum temperature during the exposure was 36 °C, increasing with a mean slope of 0.1 °C/min, and the dose rate was 5.5 kGy/h, measured with one standard deviation of 3.3%. The average total doses for the five sets of optical fibre samples were 1, 5, 15, 30, 50 kGy, respectively, with one standard deviation of 3.3%. The dosimetry system employed was of the Ethanol-Chlorobenzene (ECB) type with oscillometric readout, traceable at the National Physical Laboratory, by RISOE HDRL.

### Measurements

The BEDF samples were tested off-line for their spectral emission, e(λ), and the spectral attenuation, α(λ), before and after the irradiation. In the case of SA samples, the spectral emission was measured for both backward – e1(λ), and forward propagation – e2(λ), respectively. The doped optical fibre was also investigated as it concerns the pump losses as function of the pump power, at 830 nm. For the reported results, several pump power levels were used. The schematic of the optical power measuring points and their designation are illustrated in Supplementary Fig. S4. The pre and post irradiation tests were run at room temperature. During the laboratory investigations all the samples were kept straight, without any bending.

Supplementary Fig. S5 illustrates the setup we employed for spectral attenuation measurements. The light from a broad band lamp (WBL) is focused into the SMF @ 1310 connecting optical fiber with × 40 lens, while the output of the SMF @ 1550 is free space coupled to a monochromator (model Spex 1681) using a ×20 lens, as the optical signal passes through a filter (RG830) and is modulated by a chopper (model Standford Research SR540).

The optical signal selected by the monochromator at specific wavelengths, under the control of a PC is detected by a photodiode (InGaAs). The output of the photodiode is processed by a lock-in amplifier (LA) model SR530 (Standford Research), synchronized with the chopper frequency by the chopper controller (CC). The signal delivered by the LA is further processed by the PC which provides the spectral attenuation characteristics of the tested sample. The chopper frequency used was 133 Hz and the lock-in amplifier setting for t_pre_ and t_post_ was 0.3 s and 0.1 s, and its sensitivity was “Normal”. The monochromators scanning range was 800 nm to 1600 nm, with a step of 2 nm. The spectral attenuation was evaluated for the spectral range 800 nm to 1600 nm.

The total pump loss (T_t_) is defined by:


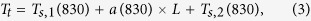


where α represents pump absorption coefficient of BEDF at 830 nm, and L is the length of BEDF sample. The tests were run at the pump wavelength of 830 nm.

The variation of the pump absorption coefficients are defined as follows:


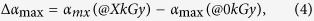



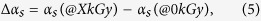



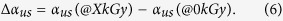


Figure of merit (M_a_) in terms of pump absorption coefficients is defined by:


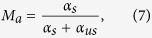


which represents a measure of the useful pump absorption α_s_ vs. the total pump absorption (α_us_ + α_us_).

The figure of merit percentage change is calculated by:


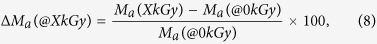


where *X* kGy = 1, 5, 15, 30, 50 kGy.

The setup for the spectral emission used is presented in Supplementary Fig. S6. The pump launching/backward detection scheme includes a LD830 laser diode module operating at 830 nm, an optical spectrum analyzer (OSA1, Model: Agilent 86140B) and a power meter (PM, Model: Newport 2823 C). The optical signal is coupled to the tested sample and the backward returned signal is directed towards the OSA through an optical fibre 810 nm/1310 nm WDM coupler. On the backward detecting side the optical signal can be switched between the OSA1 and the PM, for spectral characteristic and power measurements. On the forward detection side the output of the SMF @ 1550 optical fibre can be coupled either to another optical spectrum analyzer (OSA2, Model: Agilent 86143B) or to a PM. Both OSAs were set to a spectral resolution of 5 nm, while the sensitivity was controlled by setting the reference to −50 dBm for the backward detection, and −40 dBm for the forward detection.

The ratios of the forward emission at specific wavelength to the forward emission at a reference wavelength, used for data analysis and represented in Figs 7, 8, 9, are defined as follows:


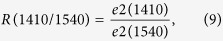



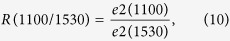



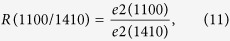


calculated from the forward emission at λ = 1100 nm, 1410 nm, and 1540 nm, respectively. As emission at 1100 nm, 1410 nm and 1540 nm are linked to the BAC-Al, BAC-Si and Er^3+^, so the ratio R(1410/1540), R(1100/1540) and R(1100/1410) under the similar pumping condition can represent the relative concentration of BAC-Al, BAC-Si and Er^3+^ to some degree.

The relative changes of these quantities are calculated according to:


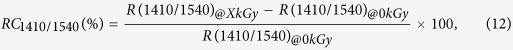



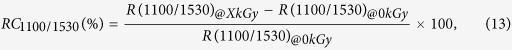



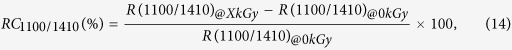


where *X* = 1, 5, 15, 30, 50 kGy and 0 kGy denotes the non-irradiated sample. RC_1410/1540_, RC_1100/1530_ and R_1100/1410_ represents the relative change of the concentrations of BAC-Si vs Er^3+^, BAC-Al vs Er^3+^, and BAC-Al vs. BAC-Si due to the radiation.

## Additional Information

**How to cite this article**: Sporea, D. *et al*. γ irradiation induced effects on bismuth active centres and related photoluminescence properties of Bi/Er co-doped optical fibres. *Sci. Rep.* **6**, 29827; doi: 10.1038/srep29827 (2016).

## Supplementary Material

Supplementary Information
