# Supplementary material for: γ irradiation induced effects on bismuth active centres and related photoluminescence properties of Bi/Er co-doped optical fibres
Source: Sci Rep. 2016 Jul 21;6:29827. doi: 10.1038/srep29827 (PMC4954960; doi:10.1038/srep29827)
Supplement: Supplementary Information [file srep29827-s1.pdf]

γ irradiation induced effects on bismuth active centres and related photoluminescence properties of Bi/Er co-doped optical fibers

D. Sporea[1*], L. Mihai[1], D. Neguţ[2], Yanhua Luo[3], Binbin Yan[3,4], Mingjie Ding[3], Shuen Wei[3], Gang-Ding Peng[3]

[1]National Institute for Laser, Plasma and Radiation Physics, Laser Metrology Laboratory, Măgurele, RO-077125, Romania

[2]"Horia Hulubei" National Institute of Physics and Nuclear Engineering, IRASM, RO-077125, Măgurele, Romania

[3]Photonics & Optical Communications, School of Electrical Engineering & Telecommunications, University of New South Wales, Sydney, NSW 2052, Australia

[4]State Key Laboratory of Information Photonics and Optical Communications, Beijing University of Posts and Telecommunications, Beijing 100876, China

*dan.sporea@inflpr.ro

Supplementary materials

Sample			SA1	SA2	SA3	SA4	SA5
Length of the BEDF (cm)			30.5	31.6	30.5	31.0	31.0
Dose (kGy)			1	5	15	30	50
	Measured parameter	Measuring conditions					
**Small signal absorption**	α@1300 nm (dB)	Before irradiation	17.8	10.9	10.8	10.6	11.4
	α@1300 nm (dB)	After irradiation	16.5	9.1	10.4	9.8	10.3
**Pump absorption**	$\acute{\alpha}_{max}$@830 nm (dB/m)	Before irradiation	58.4	55.0	56.4	53.1	52.2
	α_s@830 nm (dB/m)		45.3	41.2	40.0	36.1	32.8
	α_{us}@830 nm (dB/m)		13.2	13.8	16.4	17.0	19.4

	$\acute{\alpha}_{max}$@830 nm (dB/m)	After irradiation	72.5	71.3	77.2	86.9	91.0
	α_s@830 nm (dB/m)		51.0	41.2	40.4	44.5	46.1
	α_{us}@830 nm (dB/m)		21.5	30.1	36.7	42.3	44.9

Supplementary Table S1. The values of the attenuation related parameters measured before and after gamma irradiation.

Sample			SA1	SA2	SA3	SA4	SA5
**Forward emission**	$e_2(\lambda)$ at maximum pumping	Before irradiation	√	√	√	√	√
		After irradiation	√	√	√	√	√
	$e_2(1410)/e2(1540)$	Before irradiation	√	√	√	√	√
		After irradiation	√	√	√	√	√
		Variation	√	√	√	√	√
	$e_2(1100)/e2(1540)$	Before irradiation	√	√	√	√	√
		After irradiation	√	√	√	√	√
		Variation	√	√	√	√	√
	$e_2(1100)/e2(1410)$	Before irradiation	√	√	√	√	√

		After irradiation	√	√	√	√	√
		Variation	√	√	√	√	√
**Emission under different pumping conditions**	$e_2(\lambda)$ at different pump power	Before irradiation	√	√	√	√	√
		After irradiation	√	√	√	√	√
	$e_2(1410)$ vs pump power	Before irradiation	√	√	√	√	√
		After irradiation	√	√	√	√	√
	P_{sat}	Before irradiation	√	√	√	√	√
		After irradiation	√	√	√	√	√
	P_{sat} (after)/ P_{sat} (before)	Variation	√	√	√	√	√

Supplementary Table S2. Emission related tests performed pre and post gamma irradiation.

Measurement conditions	Sample<br>Dose (kGy)	Sample<br>Dose (kGy)	Sample<br>Dose (kGy)
	SA2<br>5 kGy	SA3<br>15 kGy	SA5<br>50 kGy
Pump power before irradiation (mW)	27.3	30.7	44.1
Pump power after irradiation (mW)	29.1	35.7	44.1

Supplementary Table S3. The pump power for emission tests - before and after the 5 kGy, 15 kGy, 50 kGy dose irradiations.

Sample name	Sample constituents	Total irradiation dose (kGy)
SA-1	SMF@1300 (1.2 m) + BEDF (30.5 cm) + SMF@1550 (2 m)	1
SA-2	SMF@1300 (1.2 m) + BEDF (30 cm) + SMF@1550 (2 m)	5
SA-3	SMF@1300 (1.2 m) + BEDF (31.6 cm) + SMF@1550 (2 m)	15
SA-4	SMF@1300 (1.2 m) + BEDF (30 cm) + SMF@1550 (2 m)	30
SA-5	SMF@1300 (1.2 m) + BEDF (30.5 cm) + SMF@1550 (2 m)	50

Supplementary Table S4. The investigated samples and the irradiation conditions.

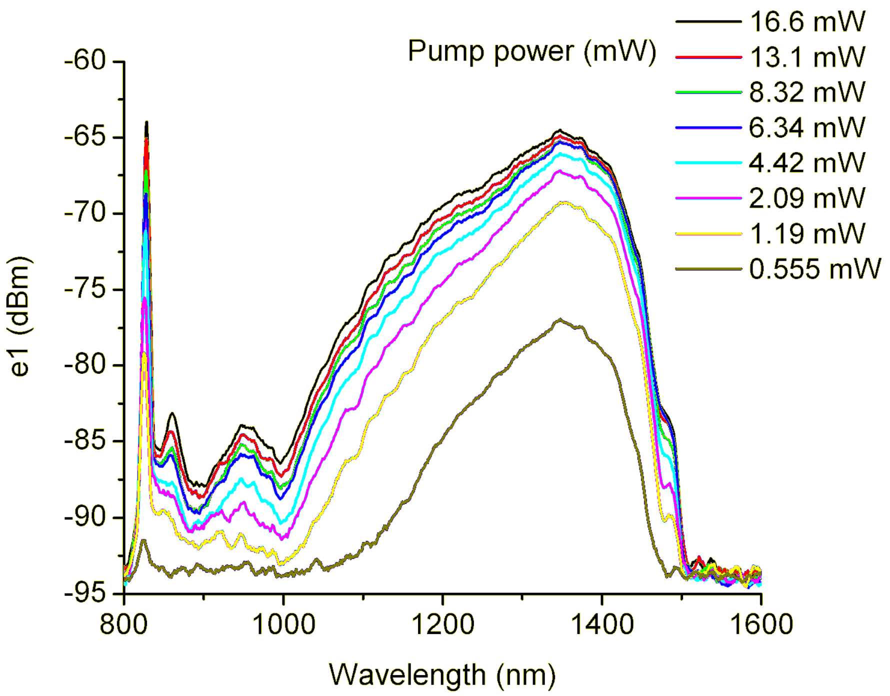

Pump power (mW)
16.6 mW
13.1 mW
8.32 mW
6.34 mW
4.42 mW
2.09 mW
1.19 mW
0.555 mW
e1 (dBm)
-60
-65
-70
-75
-80
-85
-90
-95
800
1000
1200
1400
1600
Wavelength (nm)

a

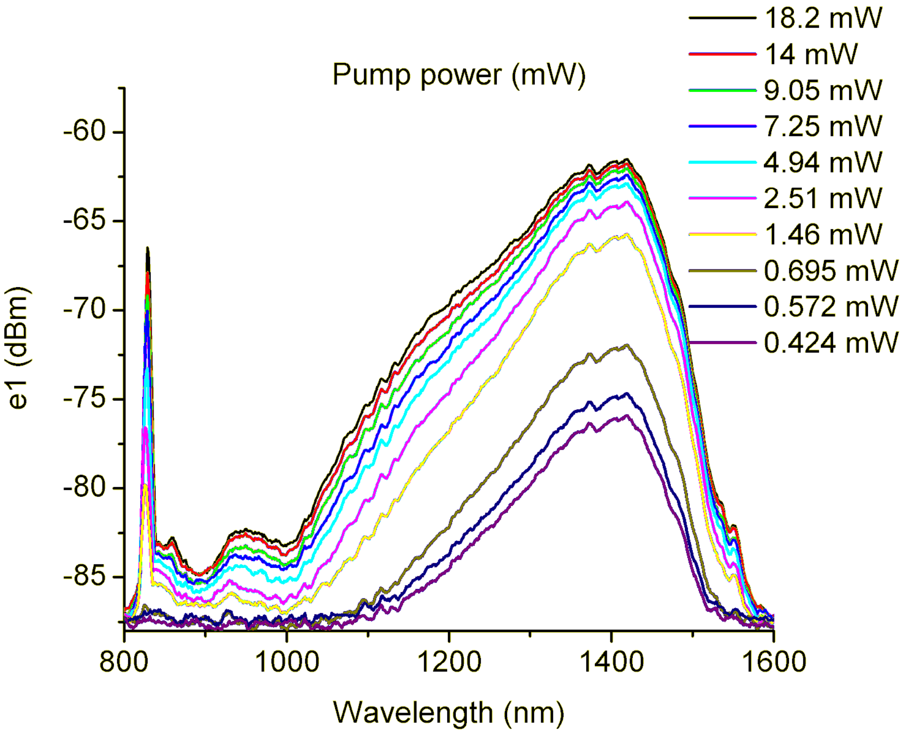

Pump power (mW)
18.2 mW
14 mW
9.05 mW
7.25 mW
4.94 mW
2.51 mW
1.46 mW
0.695 mW
0.572 mW
0.424 mW
e1 (dBm)
-60
-65
-70
-75
-80
-85
800
1000
1200
1400
1600
Wavelength (nm)

b

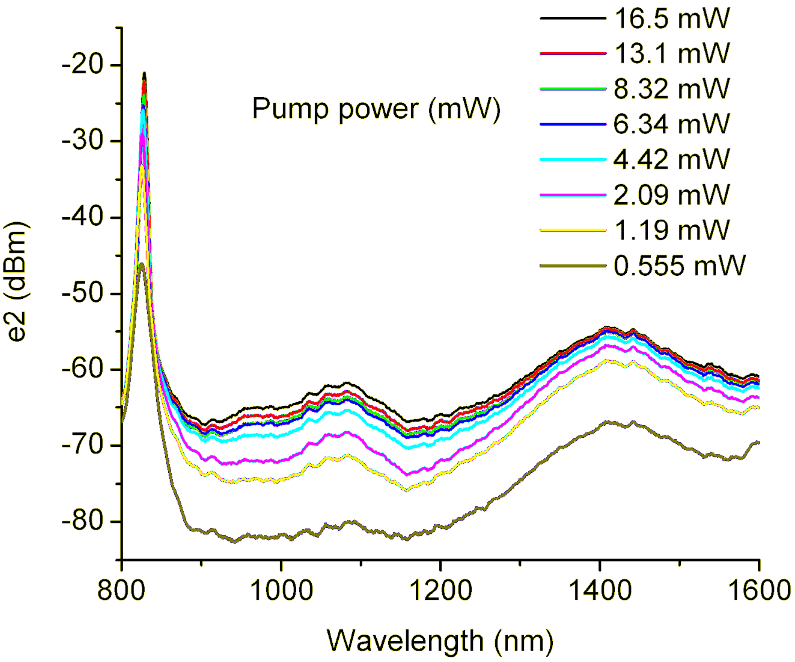

c

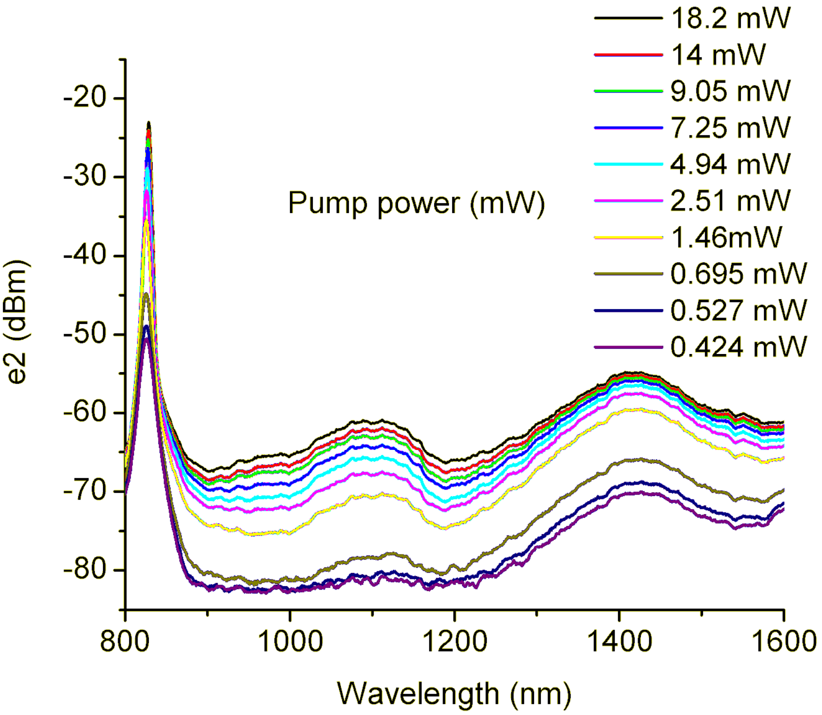

d

Supplementary Figure S1. The dependence on the pump power of: the backward $e_1(\lambda)$ propagated spectral emission for sample SA1 before (a) and after (b) 1 kGy gamma irradiation; the forward $e_2(\lambda)$ propagated spectral emission for sample SA1 before (c) and after (d) 1 kGy gamma irradiation, pump power at 830 nm.

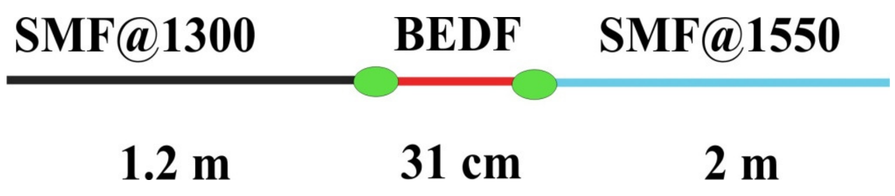

Supplementary Figure S2. The sketch of the SA optical fiber sample.

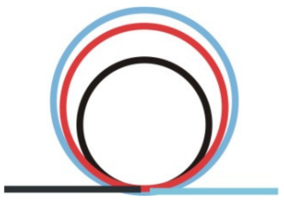

Supplementary Figure S3. The sample geometry during the irradiation

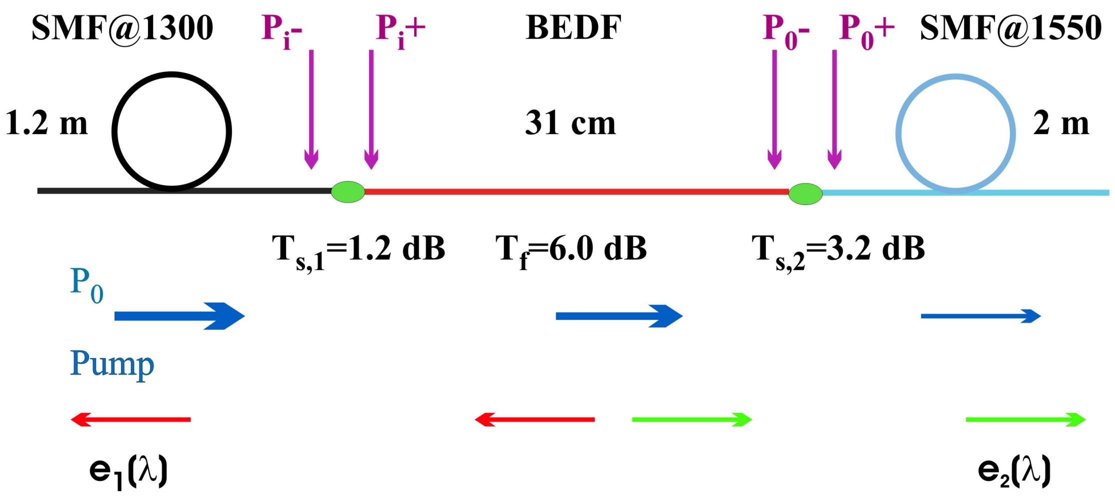

Supplementary Figure S4. The optical power measuring points for the spectral emission of the tested sample SA5 (50 kGy), used for investigations done before and after the irradiation: P_0 – the pump power; P_{i-} - the optical power at the input of the splicer coupling the SMF@1300 to BEDF; P_{i+} - the optical power at the output of the splicer coupling the SMF@1300 to BEDF; P_{0-} - the optical power at the input of the splicer coupling the BEDF to the SMF@1550; P_{0+} - the optical power at the output of the splicer coupling the BEDF to the SMF@1550; $T_{s,1}$ – transmission losses in the splicer coupling the SMF@1300 to the BEDF; $T_{s,2}$ - transmission losses in the splicer coupling the BEDF to the SMF@1550; T_f - transmission losses in the BEDF.

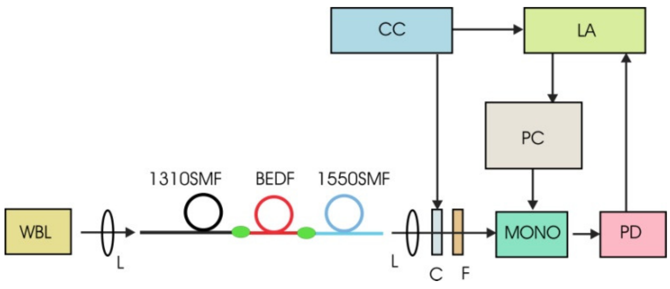

Supplementary Figure S5. The setup for spectral attenuation evaluation: WBL – wide band lamp; L – lens; F – filter; C –copper; CC – chopper controller; LA – lock-in amplifier; MONO – monochromator; PD – photodiode; PC – desk top computer.

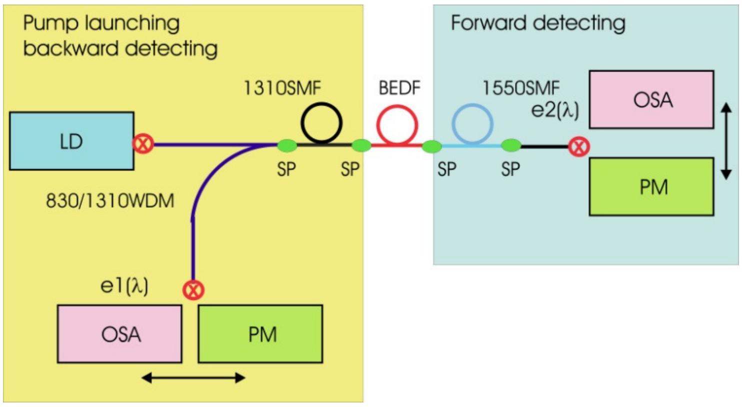

Supplementary Figure S6. The setup for spectral emission measurements: LD – laser diode; OSA – optical spectrum analyser; PM – power meter; SP – splice.
